# *Streptococcus dysgalactiae*—Contagious or Environmental?

**DOI:** 10.3390/ani10112185

**Published:** 2020-11-22

**Authors:** Nicole Wente, Volker Krömker

**Affiliations:** 1 Department of Bioprocess Engineering and Microbiology, University of Applied Sciences and Arts Hannover, D-30453 Hannover, Germany; Nicole.Wente@hs-hannover.de; 2 Department of Veterinary and Animal Sciences, Faculty of Health and Medical Sciences, University of Copenhagen, Grønnegårdsvej 2, 1870 Frederiksberg C, Denmark

**Keywords:** *Streptococcus dysgalactiae*, PFGE, contagious, environmental

## Abstract

**Simple Summary:**

The usual routes of transmission of *Streptococcus dysgalactiae* in the development of bovine mastitis are unclear. For the control of mastitis in dairy practice, improved knowledge about the transmission of this pathogen would be very helpful. The variety of strain within a herd can be used to describe its transmission behavior. Isolates of *Strep. dysgalactiae* were collected from clinical mastitis samples on different farms, and the strains were typed using a molecular method. Overall, we performed strain typing on isolates from 16 farms in Germany and found signs of the contagious transmission of *Strep. dysgalactiae* on all the farms. We observed a variety of outcomes, from a single strain in all six *Strep. dysgalactiae* cases recorded on one farm, to five strains in six cases recorded on another farm.

**Abstract:**

*Streptococcus dysgalactiae* is among the most important pathogens causing bovine mastitis. Unfortunately, there is presently a lack of clear knowledge about the mode of transmission—contagious or environmental—of this pathogen. To obtain more information on this, knowledge of the genetic diversity of the isolated microorganisms at the farm level can be useful. To observe the strain variety in different herds of cattle, isolates of *Strep. dysgalactiae* were collected from clinical mastitis samples at different farms, and the strains were typed using the pulsed-field gel electrophoresis (PFGE) method. Overall, we performed strain typing on 93 isolates from 16 farms in Germany and used an index to describe the degree of contagiosity of *Strep. dysgalactiae* at each farm. This index (CI) represents the number of isolates divided by the number of strains found in mastitis milk of clinical cases within a period of 14 months. The results differed between the farms. In one farm, all six *Strep. dysgalactiae* cases that occurred during the study period were caused by a single strain (CI = 6), while in another farm the six cases that occurred were caused by five different strains (CI = 1.2). All other farms fell between these two extremes. This indicates that *Strep. dysgalactiae* infections can occur via several routes of transmission. At the farm level, strain comparisons are necessary to determine the routes of transmission. Two strains were able to survive on the farm for a minimum of 14 months.

## 1. Introduction

Clinical mastitis (CM) in dairy cows is a common disease that significantly hampers the sustainability of milk production, as it is associated with a high consumption of antibiotics and significant economic losses [1]. In Germany, *Streptococcus* (*Strep.*) *dysgalactiae* (2.8–5.5% of all clinical mastitis cases) is the second most frequently encountered *Streptococcus* in mastitis, behind *Strep. uberis* (29–30.5% of all clinical mastitis cases) [2,3]. Understanding the epidemiology of infection of this pathogen is essential in preventing and controlling mastitis. To identify the appropriate measures in case of a high-case frequency at a farm, it is important to understand whether this pathogen is transmitted between cows or from the environment to the cow. Clear evidence on the mode of transmission of the rather contagious *Strep. dysgalactiae* does not exist so far. Some previous studies have described this species as being cow-associated [4,5], while some others have described it as being environment-associated [6,7]. In a recent Swedish study comparing isolates of *Strep. dysgalactiae* from CM samples, identical isolates were reported in several dairy herds. This supports the hypothesis that transmission occurs from farm to farm [8]. However, to control mastitis with this pathogen on an individual farm, it is not decisive whether the pathogen can basically be transmitted from farm to farm by vectors, but rather whether it shows a contagious behavior with a few different strains or a more environmentally-associated behavior with a wide diversity of strains. In the first case, the control strategies must be based on the infected animals and the milking process, while in the second case, a general improvement of the hygiene on the farm is probably more desirable. This study aimed to gain a better insight into the farm-specific transmission of *Strep. dysgalactiae* by comparing isolates from CM cases on different farms using molecular-based strain comparisons. It is assumed that a high diversity of strains within a herd indicates environmental transmission, while a low diversity of strains indicates a contagious strain behavior [9,10].

## 2. Materials and Methods

For this study, samples from farms that frequently sent their CM samples for microbiological diagnostics to the University of Applied Sciences and Arts (Hanover, Germany) were utilized. Isolates of *Strep. dysgalactiae* were obtained from quarter milk samples collected by nonstudy staff veterinarians or farmers who had previously identified the animals with clinical signs of mastitis. All sizes and types of farms (such as organic and conventional) were included in the study, provided they delivered a minimum of two *Strep. dysgalactiae* isolates from two CM cases. To exclude the persistent mastitis cases, the cases were restricted to one *Strep. dysgalactiae* isolate per animal with CM. Isolates collected over 14 months were considered.

Microbiological diagnostics were performed following the recommendations of GVA [11]. About 10 µL of each sample was cultured on a quadrant of an esculin blood agar plate (Oxoid, Wesel, Germany) at 37 °C for 48 h. Isolates of *Strep. dysgalactiae* were Gram-positive, catalase- and esculin-negative cocci belonging to the Lancefield serotype C (Diamondial Strep Kit, Diamondial, Sées, France). All the isolates were stored in Brain Heart Infusion Broth (BHI) (Merck, Darmstadt, Germany) supplemented with 20% anhydrous glycerol (Merck, Darmstadt) at −80 °C until further analysis.

To compare all the collected isolates within one herd, Pulsed Field Gel Electrophoresis (PFGE) was performed. For this purpose, DNA was extracted from the collected isolates using a Bio-Rad CHEF Genomic DNA Plug Kit (BioRad, Munich, Germany). The obtained DNA was then digested with 20 U of SmaI (New England Biolabs, Frankfurt am Main, Germany) at 25 °C for 2 h. For the separation of the resultant DNA fragments, a Clamped Homogeneous Electric Fields Dynamic Regulation System with a performing angle of 120° (CHEFDR II System, Bio-Rad, Munich, Germany) was used and filled with 0.5× TBE buffer continuously chilled to a temperature of 14 °C. The system was programmed for pulse timings of 1 to 15 s for 11.5 h and of 15 to 45 s for 13.5 h at 5 V/cm (210 V). The lambda PFG ladder (48.5–1018 kb; New England Biolabs, Ipswich, MA, USA) was run in each applied gel. The gel was stained with Midori Green Advanced (Nippon Genetics Europe, Düren, Germany) and visualized using the InGenius gel documentation system (Syngene, Cambridge, UK). Isolates with identical restriction patterns were identified as identical strains.

To evaluate the contagiousness of *Strep. dysgalactiae* on a farm, an index called the Contagiousness Index (CI) was implemented, which puts the number of identified strains in relation to the number of isolates. This index represents the number of isolates divided by the number of strains. If the number of isolates equals the number of strains, the CI is 1, indicating a low contagiousness of the *Strep. dysgalactiae* in a farm; on the other hand, a growing CI is influenced by a low diversity of the strains and amount of each strain, which indicates a higher contagiousness of *Strep. dysgalactiae* within a farm.

The results of the investigations are presented descriptively. Simple statistics, such as a Chi-square test, were used to compare the management type, size, and location. All statistical analyses were performed with SPSS 26.0 (IBM SPSS 26.0, Armonk, NY, USA).

## 3. Results

In total, 16 farms were involved in this study. Two farms were organic, and 14 were conventional. Four farms had less than 100 cows, and 12 had more. Seven farms were in the north of Germany, five in the Central Region, and four in eastern Germany. The criteria (management type, size, and location) were not associated with the CI (*p* > 0.05). All 93 isolates were analyzed by the PFGE method (Table 1). A maximum of seven strains (farm G, CI = 2) and a minimum of one strain (farm A, CI = 6) were recorded per farm during the study period. The lowest CI was 1.2 (farm P), and the highest was 6 (farm A). A minimum of two *Strep. dysgalactiae* CM cases were reported to have been caused by the same strain, and, consequently, no farm could achieve a CI of 1.

## 4. Discussion

Due to the study design, the observed numbers of isolates were highly influenced by farmers’ skills in detecting a CM and their willingness to collect milk samples from the detected cases. Subsequently, no subclinical *Strep. dysgalactiae* infections could be considered, and the existing status of microbial infection of the entire herd was therefore unknown. During the trial period in this study, the effect of an intervention by the veterinarian or the farmer, based on the detection of the *Strep. dysgalactiae* in the herd, should not be sidelined. Different herds underwent different treatments for *Strep. dysgalactiae* mastitis. Due to the low minimal inhibitory concentration (MIC) values of *Strep. dysgalactiae* [12], a bacteriological cure is highly likely, but on the other hand its ability to interact with several plasma proteins and extracellular host-derived proteins, produce hyaluronidase and fibrinolysin, and survive within the mammary epithelial cells without damaging them [13] can, along with its persistence [3,14], be the reason for its extended survival period within a herd. Pathogens with special characteristics (e.g., adaptation to survive within the host, transmission during milking process) may dominate at the herd level and therefore appear contagious [10,15,16]. A low variety of strains could also be caused by a contagious strain and/or by an environmental hotspot harboring a high concentration of the strain.

PFGE was used for a strain comparison in this study. This method has a high discriminatory potential and has been used as a gold standard [17]. To achieve a stricter consideration of infectious strains, only identical PFGE patterns, and not highly related strains, were defined as identical strains. In this respect, the detection of identical strains at the herd level indicates a predomination of the strains. Nonetheless, as long as no clonal characterization is performed, the infection of animals with identical *Strep. dysgalactiae* PFGE profiles from different sources is possible and, consequently, cannot be completely excluded. On the other hand, the detection of different strains in each animal by the PFGE ensures an environmental transmission [10].

Since we took one *Strep. dysgalactiae* isolate per animal, each cow was taken into account only once. Consequently, there was no possibility of gaining an identical strain out of a persistent infection several times. However, this strategy leads to a lack of information on reinfections with the same or another strain and, therefore, probably hampers the information on various strains within a farm. Consequently, the bacteriological cure could not be investigated due to the study design, and information on reinfection could not be collected in any manner. Therefore, we cannot answer the question of whether recurrent mastitis with *Strep. dysgalactiae* is caused by persistent strains or by other strains. The aim of our work was not a complete analysis of infection dynamics with *Strep. dysgalactiae*. Rather, the aim was to answer the question of whether there was a low or a high diversity of *Strep. dysgalactiae* strains on dairy farms in order to be able to choose the right control strategies.

The CI changes due to the frequency of the *Strep. dysgalactiae* appearance on the farm. The appearance in our study equals the number of *Strep. dysgalactiae*-positive samples that we gained from the farms. The latter definitely depends on the farmer’s willingness to take a sample. The larger the number of isolates from a dairy farm, the more reliable the statement about the contagiousness of the strains in the dairy farm. In this respect, the CI also allows an assessment of the reliability of the statement. However, this remains a relative measure of the contagiousness of *Strep. dysgalactiae* at the herd level.

A very large recent epidemiological study has shown that *Strep. dysgalactiae* causes, on average, 55.0% mild, 38.7% moderate and 6.3% severe mastitis. Half of clinical mastitis occurs in the first 100 days of lactation. After therapy, the bacteriological cure rates of clinical cases with this pathogen were 82.9% [18]. Another recent study illustrated that subclinical infections with *Strep. dysgalactiae* had particularly high excretion rates compared to other mastitis pathogens [19]. If the clinical virulence of *Strep. dysgalactiae* is measured by its contagiousness, it appears to be very heterogeneous between the different strains. Bacteriological cure rates, common prevalences of subclinical and clinical infections, and clinical expressions all indicate that the virulence of *Strep. dysgalactiae* in bovine mastitis is somewhat lower than that of *Strep. uberis*.

The investigated *Strep. dysgalactiae* strains never showed a completely heterogeneous picture at the farm level, indicating a completely environmentally associated transmission picture. In varying degrees of frequency, individual strains dominated at the farm level, which is mostly seen as a sign of contagious transmission or transmission from hotspots. In three of the investigated farms, the detection of *Strep. dysgalactiae* as a contagious pathogen had to be evaluated, as all the identified mastitis cases in these farms were caused by only one strain. These results indicate that the infection behavior of *Strep. dysgalactiae* is farm-specific, which makes it useful to conduct a strain comparison before the implementation of specific prophylactic measures. Further investigations with more dairy farms are necessary in order to confirm the classification of *Strep. dysgalactiae* as a mastitis pathogen.

## 5. Conclusions

*Strep. dysgalactiae* can occur as either a cow-associated or environmentally associated mastitis pathogen. As shown in our study, cow-associated/contagious strains are present on every dairy farm.

The results suggest that a comparison of strains at the herd level on dairy farms with mastitis *Strep. dysgalactiae* could be useful in selecting appropriate control measures. Furthermore, the results allow the assumption that strains of *Strep. dysgalactiae* may persist on dairy farms for more than one year.

## Figures and Tables

**Table 1:** The number of isolates and strains of *Streptococcus dysgalactiae* on each farm.

Farm	CI	1. Strain	2. Strain	3. Strain	4. Strain	5. Strain	6. Strain	7. Strain	Ʃ = 93
A	6	6							6
B	5	5							5
C	4	4							4
D	3	5	1						6
E	2.3	4	2	1					7
F	2	5	2	2	1	1	1		12
G	2	4	4	2	1	1	1	1	14
H	1.6	2	2	1					5
I	1.5	2	1						3
J	1.5	2	1						3
K	1.5	2	1						3
L	1.5	2	1						3
M	1.5	3	1	1	1				6
N	1.5	2	2	1	1				6
O	1.3	2	1	1					4
P	1.2	2	1	1	1	1			6

A wide variety of strains was recorded in farms F and G ((Appendix A) Table A1). However, farms A, B, and C showed a low variety of strains, which persisted over a long period within the farms (Table A1). Two strains, C1 and E1, were able to survive on the farm for a minimum of 14 months.

## Appendix A

**Table A1:** *Streptococcus dysgalactiae* strains isolated from CM milk samples over 14 months.

		Months *													
Farm	Strain	1st	2nd	3rd	4th	5th	6th	7th	8th	9th	10th	11th	12th	13th	14th
A	A 1	• ***	•••		•						•				
B	B 1	••	•							•			•		
C	C 1	•							•						•
D	D 1	•	•••		•										
	D 2											•			
E	E 1	•													•
	E 2							•				•		•	•
	E 3														•
F	F 1	•		•											
	F 2	•													
	F 3		•												
	F 4			•	••				••						
	F 5				•		•								
	F 6							•							
G	G 1	•	•			•			•						
	G 2				•		•	•			•				
	G 3					•									
	G 4							•	•						
	G 5							•							
	G 6										•				
	G 7											•			
H	H 1	•		•									•		
	H 2				•		•								
	H 3					•									
I	I 1	•			•										
	I 2				•										
J	J 1	•													
	J 2		•		•										
K	K 1	••													
	K 2		•												
L	L 1	•		•											
	L 2	•													
M **	M 1	•••													
	M 2	•													
	M 3	•													
	M 4	•													
N	N 1	•			•										
	N 2		•												
	N 3													••	
	N 4														•
O	O 1	•													
	O 2		•	•											
	O 3					•									
P	P 1	•													
	P 2		•												
	P 3				•										
	P 4										•			•	
	P 5												•		

* Months since the first *Strep. dysgalactiae* isolate was recorded at a farm; ** samples that arrived at once. *** one dot stands for one isolate.
